# Practical Gynæcology

**Published:** 1879-04

**Authors:** 


Practical Gynæcology. A Handbook of the Diseases of Women. By Haywood Smith, M.A. M.D., Oxon, Member of the Royal College of Physicians, etc., with Illustrations. Lindsay & Blakiston, Philadelphia. 1878.

Several new works that should have been noticed earlier have been neglected, perforce, on account of being crowded out by circumstances necessarily urgent. Among the number is this neat little volume of two hundred and odd pages, which is, as it purports to be, a splendid practical and compendious expositor of the diseases peculiar to women. Each disease is treated of briefly and yet explicitly, in a manner that renders the work of almost inestimable value as a book of reference.

For instance mucous polypus is given with its definition, causes, symptoms, signs, diganosis, prognosis and treatment. Many cuts and drawings are introduced illustrative of parts that otherwise might be obscure.

